# Household Energy Insecurity and COVID-19 Have Independent and Synergistic Health Effects on Vulnerable Populations

**DOI:** 10.3389/fpubh.2020.609608

**Published:** 2021-01-21

**Authors:** Godfred O. Boateng, Laura M. Phipps, Laura E. Smith, Frederick A. Armah

**Affiliations:** ^1^Department of Kinesiology, College of Nursing and Health Innovations, The University of Texas at Arlington, Arlington, TX, United States; ^2^Department of Epidemiology and Environmental Health, School of Public Health and Health Professions, University at Buffalo, Buffalo, NY, United States; ^3^Department of Environmental Science, University of Cape Coast, Cape Coast, Ghana

**Keywords:** COVID-19, energy insecurity, household, health effects, health inequity, vulnerable populations

## Abstract

Household energy insecurity (HEINS) is detrimental to the health of the poor and most vulnerable in resource-poor settings. However, this effect amidst the COVID-19 pandemic and the uneven implementation of restrictions can create a synergistic burden of diseases and health risks for the most vulnerable in low- and middle-income countries, exacerbating the health equity gap. Based on existing literature, this paper develops three key arguments: (1) COVID-19 increases the health risks of energy insecurity; (2) HEINS increases the risk of spreading COVID-19; and (3) the co-occurrence of COVID-19 and HEINS will have compounding health effects. These arguments make context-specific interventions, rather than a generic global health approach without recourse to existing vulnerabilities critical in reducing the spread of COVID-19 and mitigating the effects of energy insecurity. Targeted international efforts aimed at financing and supporting resource security, effective testing, contact tracing, and the equitable distribution of vaccines and personal protective equipment have the potential to ameliorate the synergistic effects of HEINS and COVID-19 in resource-poor countries.

## Introduction

Energy insecurity, the lack of access to adequate, affordable, reliable, acceptable, and clean sources of energy for a healthy and sustainable livelihood, has detrimental consequences for many households in developing countries (1, 2) and low-income settings in the developed world (3). Household energy insecurity (HEINS), like other resource insecurities such as food and water insecurity, can be symptomatic of broader economic disadvantages and social inequalities faced by the poor. The effects of energy insecurity at the household level are numerous and can pose psychosocial (e.g., stress, anxiety), economic (e.g., decrease in income generating activities), disease (e.g., diarrhea, respiratory infections), and nutritional burdens (e.g., dietary restrictions, food insecurity) on the most vulnerable (1, 3, 4). These burdens are hypothesized to worsen as a result of the coronavirus disease (COVID-19), which is caused by severe acute respiratory syndrome coronavirus 2 (SARS-CoV-2), due to the uneven implementation of restrictive health mandates. Measures put forth by the World Health Organization (WHO) that focus on total lockdowns (5), in particular, may increase the disease burden associated with energy insecurity at the household level. This supports the fact that a generic public health approach to mitigating infectious disease may not be effective in certain settings (6). HEINS can also increase the spread of COVID-19 in most low-income households due to the need for their members to leave home to collect household fuels, food, and water resources. The co-occurrence of COVID-19 and HEINS could amplify the psychosocial and disease burdens experienced by low-income households and further widen the health equity gap. This paper aims to draw out the independent effects of HEINS and COVID-19 and discuss their potential syndemic effects on households.

## COVID-19 Increases the Health Risks of Energy Insecurity

COVID-19 has proven difficult to control as effective and safe vaccines are still out of reach of the very poor (7, 8). By the end of June 2020, WHO reported almost 10 million cases of infection and half a million deaths (9). The WHO report suggested the cases were doubling about every 6 weeks, with sub-Saharan Africa (SSA) expected to be the next epicenter of the disease. WHO cautions national and local authorities to balance interventions when addressing the health impact of COVID-19 citing unintended consequences caused by uneven interventions, such as loss of income or the interruption of essential services (9). Measures aimed at limiting the spread of the virus from one area to another include, among other things, limiting the movement of persons locally or nationally. These preventive measures, however, may have unintended consequences (5). While there is evidence to show that sheltering-in-place reduces the risk of COVID-19 infection, the implementation of this generic approach, which has taken the form of curfews and partial lockdowns in some LMICs, without recourse to existing vulnerabilities has created worse conditions for many households.

Aside from psychosocial effects such as anxiety, stress, depression, frustration, and the increased tendency to attempt or commit suicide (10, 11), having to stay indoors has increased energy insecurity for many households in sub-Saharan Africa. The World Energy Outlook 2020 report indicates the pandemic has reversed progress made in sub-Saharan Africa, with the number of those lacking electricity increasing by 13 million people, or 2%, from last year to more than 590 million in 2020 (12). A number of factors has led to this increase. Primarily, utilities that deliver clean energy now face serious financial constraints, African governments have now been compelled to shift their immediate priorities to securing the health of their populations, and there is limited financial aid available to expand and improve electricity infrastructure. Additionally, the International Energy Agency estimates that the rise in poverty levels worldwide has made basic electricity services unaffordable for over 100 million people with electricity connections, resulting in households reverting to more polluting, and inefficient sources of energy (12). In 2017, the International Energy Agency report on transitions from poverty to prosperity showed that about 2.8 billion people lacked access to clean cooking, with 2.5 billion relying on the traditional use of solid biomass (13). This number is estimated to be increasing due to the COVID-19 pandemic as more households' resort to solid fuels. Solid biomass is sourced from materials such as wood, straws, agricultural residue, process waste (sawdust), algae, and seaweed. The affordability and availability of biomass have made it a primary source of cooking fuel for most low-income households in developing countries in Asia and sub-Saharan Africa. However, social and health interventions such as total or partial lockdown aimed at reducing the spread of COVID-19 has also hampered access to energy resources and other developmental programs to secure clean energy, resulting in debilitating consequences on the health of many low-income households.

The indirect and social effects of the pandemic are predicted to have worse consequences on women than men; in fact, the United Nations Population Fund warns that as nations implement shelter-in-place measures, women are more likely to bear higher proportions of the domestic burden (14). In this context, women who cook with solid fuels indoors are at higher risk of respiratory infections during the lock down as they are increasingly exposed to poisonous gases from incomplete fuel combustion (15). Households that are energy insecure may also be in danger of food poisoning and gastrointestinal infection due to the consumption of contaminated food and water that could not be sufficiently heated (1). Furthermore, the absence of cooking fuel in households whose main food sources are staples that require cooking could lead to hunger caused by lower caloric intake, malnutrition, and associated health complications. It could also lead to an increase in the anxiety, stress, and frustrations associated with energy insecurity (1). Additionally, energy insecurity may lead to the termination of income-generating activities (e.g., dressmaking, hairdressing, and commercial cooking) for households that depend on energy for sustainable livelihood. Consequently, minimizing the transmission of COVID-19 in developing countries calls for specific interventions and smart policies that consider the complex conditions in which people live.

## Energy Insecurity Increases the Risk of Spreading COVID-19

A bidirectional relationship is hypothesized to exist between energy insecurity and the spread of COVID-19. For instance, whereas energy-secure households have ready access to energy and may have the capacity to remain home when required, members of energy-insecure households in developing countries often must leave home to gather firewood or other resources for energy and may consequently have a higher risk of being exposed to the coronavirus (16). This may further be exacerbated by the depletion of existing fuel stalks as entire households' shelter-in-place and the frequency of cooking increases. We further contend that energy insecurity could impede adherence to guidelines and increase the spread of COVID-19 not only in developing countries but also in low-income settings within developed countries.

Economic deprivation in these low-income settings compounded by poor housing worsens the effects of energy insecurity leading to an increased risk of exposure to COVID-19. Hernández (3) and Hernández et al. (17) report that poor housing quality and lack of proper maintenance leads to poor insulation, limited ventilation, and the malfunctioning of heating and cooling systems. Sheltering-in-place in such conditions further exacerbates the health risks associated with poor housing, such that in the United States, counties with a higher percentage of households with poor housing have been found to have a higher incidence of morbidity and mortality associated with COVID-19 (18). Second, it means low-income households will be less likely to adhere to guidelines supporting social distancing measures. Leaving the house for part or the entire day might mitigate the effects of energy insecurity within the house but will increase the risk of COVID-19 infection for such populations. The lack of cooling systems in households amidst humid temperature and heat waves can mean an increase in visits to cooling centers, which are commonly implemented in hot regions within developed countries to provide a safe location to low-income populations from heat-related morbidity and mortality (19). Daanen et al. (20) assert that going to public cool areas in the hot season interferes with stay -at-home recommendations to reduce the spread of the virus. For households bound by stay-at-home orders, constraints on movement mean energy-insecure households in cold climates may have to continue to live in the cold with an increased risk of hypothermia, while those that live in hot and humid climates have an increased risk of hyperthermia. The need for energy security may also necessitate some low-income household members to leave their housing to seek employment, increasing their risk of infection. These findings from prior studies call for an investigation into how poor housing and energy insecurity may be exacerbating the spread of COVID-19 infections among low-income households and increasing the health equity gap.

## The Syndemic Effects of COVID-19 and Energy Insecurity

Syndemic theory posits that diseases cluster due to the interaction between social, environmental or biological, and political factors, which have compounding effects on each other (21). Using this framework, it is possible to see the co-occurrence of COVID-19 and energy insecurity exacerbating independent effects on each other and compounding the health effects associated with these biological and social forces. Thus, it is possible to see a significant increase in morbidity and mortality where COVID-19 interacts with social determinants of health. Household energy insecurity can result in low-income households burning biomass for heat and cooking, leading to household air pollution and subsequent respiratory diseases (COPD), acute lower respiratory infections in children and lung diseases in adults (22). In developed countries, energy insecurity at the household level can result in alternative heating behaviors such as using ovens to burn trash and turning on space heaters (e.g., wood stoves) and other heating equipment (e.g., fireplaces/chimneys, central heat, water heaters, and heat lamps) (23). The use of such heating fires has been found to be the leading cause of home fires and responsible for 86% of deaths and 78% of injuries (23, 24). Similarly, COVID-19 has been found to increase the risk of lung complications such as pneumonia, acute respiratory distress syndrome, and sepsis. In both co-occurring events, symptoms such as shortness of breath, fever, and cough are common. Therefore, we argue that the occurrence of COVID-19 with HEINS-related diseases will increase adverse health effects for such households. This positions households in LMICs at a much higher risk of death from the disease than those in the developed countries. Secondly, dealing with energy insecurity and a total lockdown in the bid to prevent the spread of COVID-19 can decrease household income available for electricity or wood fuel and exacerbate the effects of energy insecurity (25). The syndemic effects of a pandemic and energy insecurity can subject the poorest and most vulnerable populations to both indirect health effects (discontinuation of essential health services, economic fall-out, social isolation) and to the disease itself, due to increased exposure (i.e., lack of hygiene, sanitation, ventilation, and preventive measures) and the presence of underlying conditions (25). In this context, both biological and socio-economic forces create not just a double burden, but a multiple burden of diseases on the most vulnerable in society, exacerbating the health equity gap. An in-depth investigation is needed to provide evidence of these syndemic effects and the associated health outcomes.

## Discussion and Implications

This paper advances arguments pertaining to the independent effects of household energy insecurity and COVID-19 and their synergistic effects on vulnerable populations in poor resource settings. Although these propositions call for additional research, they have implications for the current approach implemented in low-income countries, which must prevent the spread of COVID-19 while mitigating the adverse effects of HEINS. The fact that the majority of households in poor resource settings do not have in-house access to energy resources suggests that broad preventive measures by WHO might not be entirely effective in developing countries within Asia or SSA, making countries in these two geographic areas highly vulnerable to rapid intra-country spread of COVID-19. With WHO predicting SSA as the next epicenter, it is important for global health experts, WHO and African leaders to develop context-specific interventions that also address the challenges intrinsic to HEINS. In addition, as safe COVID-19 vaccines become available, it is critical that countries in poor resource settings are supported by the international community to procure these vaccines for their populations. Indeed, adherence to a global equitable access to COVID-19 vaccines may be one of the many pathways to improving resource security for the poor. Targeted international efforts aimed at financing and supporting resource security (energy, water, and food resources), effective testing, contact tracing, and the equitable distribution of vaccines and personal protective equipment have the potential to ameliorate the synergistic effects of HEINS and COVID-19. Next, we echo suggestions made by Dahab et al. (26) that in low-income countries, time-limited movement restrictions should be considered primarily as an opportunity to develop sustainable and resource appropriate mitigation strategies. We add that resources essential to daily living and survival should be made available and accessible in poor resource settings. Finally, the compounding effects of COVID-19 and energy insecurity have the potential to adversely affect the health of vulnerable populations, mandating the need for a more inclusive and proactive approach in dealing with the spread of COVID-19 in LMICs.

## Data Availability Statement

The original contributions presented in the study are included in the article/supplementary material. Further inquiries can be directed to the corresponding author.

## Author Contributions

GB conceptualized, wrote, and revised the manuscript. LP, LS, and FA contributed to writing and critical revision of the manuscript. All authors approved the final version of the manuscript for publication.

## Conflict of Interest

The authors declare that the research was conducted in the absence of any commercial or financial relationships that could be construed as a potential conflict of interest.

## Abbreviations

COVID-19: Coronavirus diseases
HEINS: household energy insecurity
IEA: International Energy Agency
LMIC: low-and middle-income countries
SARS-CoV-2: severe acute respiratory syndrome coronavirus 2
SSA: sub-Sahara Africa
WHO: World Health Organization.
